# Does Self-Control Promote Prosocial Behavior? Evidence from a Longitudinal Tracking Study

**DOI:** 10.3390/children9060854

**Published:** 2022-06-08

**Authors:** Jingjing Li, Yanhan Chen, Jiachen Lu, Weidong Li, Shuangju Zhen, Dan Zhang

**Affiliations:** 1 School of Psychology, South China Normal University, Guangzhou 510631, China; 2020010222@m.scnu.edu.cn (J.L.); 2019022970@m.scnu.edu.cn (Y.C.); shuangjuzhen@foxmail.com (S.Z.); 2019023034@m.scnu.edu.cn (D.Z.); 2 School of Physical Education, Guangzhou University, Guangzhou 510006, China

**Keywords:** self-control, prosocial behavior, life satisfaction, friendship quality

## Abstract

Although numerous researches have shown that self-control is a significant promoter of prosocial behavior, the mechanism behind this relationship is still unclear. According to the organism–environment interaction model and self-control model, this study researched whether life satisfaction played a mediating role between self-control and adolescents’ prosocial behavior and if friendship quality played a moderating role between self-control and prosocial behavior. This study used a longitudinal tracking research (T1&T2; and the interval between T1&T2 is 6 months). A total of 1182 Chinese middle school students participated the survey. They were between 12 and 15 years old (average age: 14.16 years old, SD = 1.29). Results indicated that life satisfaction played a mediating role between self-control and adolescents’ prosocial behavior. Furthermore, this direct relationship in the link between self-control and prosocial behavior was significant when adolescents had a good-quality friendship. These results highlight that life satisfaction plays an important role in the relationship between self-control and prosocial behavior. The present study further determined that a high-quality friendship was an important factor that amplified this direct effect.

## 1. Introduction

Living in a social environment, human beings often engage in positive behaviors that are beneficial to society, such as donating, helping others, and cooperating. These behaviors are called prosocial behaviors [1]. As an important part of individual socialization, prosocial behavior is common when interacting with others, working, and conducting other communication activities. Prosocial behavior enables individuals to maintain a good relationship with others and has a profound impact on the development of people’s lives [2,3,4]. The cultivation of an individual’s prosocial behavior is related to societal development. The study of prosocial behavior has gradually become a hot topic among psychologists and sociologists [5]. Studies have indicated that self-control [6], peer relationships [7], life satisfaction [8], and other factors are closely related to prosocial behaviors, but it is unclear how these factors have an impact on prosocial behavior. According to the organism–environment interaction theory [9], an individual’s behavior is influenced by many factors. These factors that influence individuals’ behavioral decisions may not occur independently but interact with each other. Therefore, this study explores the influence of friendship quality, life satisfaction, and self-control on promoting adolescent prosocial behavior to further understand mechanisms behind prosocial behavior and provide a basis for the cultivation and promotion of adolescent prosocial behavior.

### 1.1. Self-Control and Prosocial Behavior

As a core element in adolescent healthy development, self-control is a person’s capacity to govern ideas, emotion, impulsive behavior that ensures individuals’ inner thoughts, and external behaviors that conform to the norms of social morality and contribute to the realization of established goals [10,11] Most of the literature indicates that prosocial behavior is positively related with self-control. However, in the literature, a few authors claim the opposite [12,13,14]. Researchers have shown that highly self-controlled individuals can adapt well to the environment, have a clear plan on career development, achieve good academic performance, and experience more positive emotion and more subjective well-being [13,15]. Meanwhile, high self-control ability is closely related to prosocial tendencies, such as helping behavior and donation behavior. In contrast, low self-control ability was significantly related to anti-social behaviors, including selfishness, crime, violence, and aggression [12,14]. A person with low self-control ability is more likely to violate social rules and has difficulty in resisting temptation, therefore being more likely to act impulsively when facing problems. It is precisely because of the existence of self-control that individuals constantly examine whether their behaviors conform to social norms, regulate unhealthy emotions such as anger, and ultimately reduce the occurrence of anti-social behaviors such as crime and aggression [1,16]. To better adjust to society, individuals should overcome temporary temptations and engage in more prosocial behavior that is conformed to social and long-range objectives, such as helping others and donating [17,18]. Individuals who engage in prosocial behavior often sacrifice their interests. When individuals’ interests conflict with others’ interests, they often experience the inner conflict of “self-interest” and “altruism” [19,20]. Overcoming this type of conflict requires self-control. The self-control energy model regards self-control as a resource of depletion, and low self-control or depletion diminishes prosocial behavior [21]. Empirical research has further shown that self-control is significantly related to prosocial behavior. High self-control ability is closely associated to helping behavior, and low self-control ability or ego depletion indicates less prosocial behavior [11,22].

### 1.2. Life Satisfaction as a Potential Mediator

Life satisfaction is a person’s cognitive assessment of overall quality of life in their daily interactions [23]; it is an important indicator of positive development in adolescents [24,25]. Research has shown that life satisfaction is closely related to self-control and prosocial behavior [26,27,28]. We speculate life satisfaction plays a mediating role between self-control and adolescents prosocial behavior.

First, self-control influences an individual’s life satisfaction. Since highly self-controlled people are relatively able to adopt positive coping strategies, they are focused on satisfying psychological needs and thus perceive optimal job satisfaction levels, all of which are beneficial to their experience of a satisfactory life [26,27,28]. According to the locus of control theory [29], individuals’ control of their living environment and events is the primary motivation for behavior. Individuals with a relatively high sense of control have stronger autonomy and efficiency, and a lack of control can lead to psychological problems such as depression [30]. Moreover, high self-control is one of the protective factors of life satisfaction [31] whereas low self-control can negatively affect adolescents’ psychology health and overall well-being [32]. Furthermore, highly self-controlled individuals prefer to pursue goals with better outcomes, which in turn increases their life satisfaction [33,34]. Empirical studies further indicated self-control significantly positively predicts satisfaction in life [35,36].

Secondly, life satisfaction positively predicts prosocial behavior [37]. Individuals with high life satisfaction generate more positive emotions and face life optimistically, so it also has a positive effect on prosocial behavior. Based on broadening and building the theory of emotion [38], positive emotions expand the scope of an individual’s focus and increase their attention to all kinds of external information in a given situation. Therefore, positive emotions make it easier for people to recognize the help signals in the surrounding environment and increase the possibility of helping behavior. Secondly, positive emotions can expand the breadth of cognition [39]. Individuals with more positive emotions are more optimistic and far-sighted in their self-evaluation (their ability and resources to help others). They are more willing to lose some immediate benefits in exchange for possible future gains, so they are more inclined to engage in prosocial behaviors [40]). In addition, individuals with positive emotions are more likely to recall events with positive emotions, such as pleasant experiences of helping others or feelings of gratitude when helped by others, and pleasant experiences will promote individuals to make the decision of helping others [41].

### 1.3. Friendship Quality as a Potential Moderator

As individuals enter adolescence, they communicate more frequently with friends in their daily campus life, gradually reducing their dependence on parents and changing peer relationships [42]. Friendship is an individual-oriented, bi-directional structure, reflecting the emotional connection between two individuals characterized by trust, with persistence, intimacy, and stability [43]. Friendship quality is an important indicator of the peer relationship and a basic characteristic of friendship [44].

A large number of researches showed that interpersonal familiarity and whether an individual has appropriate and effective communication skills and strategies will affect prosocial behavior [45,46,47]. Adolescents with good peer relationships tend to show more prosocial behavior [48]. For example, the higher the degree of peer acceptance, the more motivated adolescents are to learn prosocial behaviors from their peers [49] This is because individuals with good peer relationships generally have strong social cognitive abilities, such as theory of mind [50], opinion picking [51] and empathy [52,53], and these abilities are key factors in promoting prosocial behaviors. Actually, adolescents affiliated with good peer relationships are more likely to get positive feedback from their peers, and their prosocial behaviors are further strengthened, which causes them to exhibit more prosocial behaviors.

**Figure 1:**
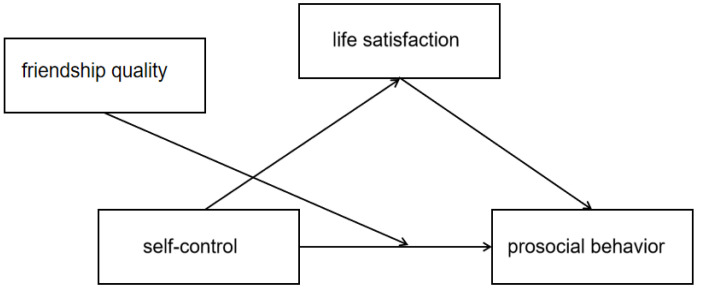
The proposed theoretical model.

Prosocial behavior sometimes requires individuals to focus on the needs of other people at the expense of personal interests, so self-control ability plays an important role in engaging in prosocial behavior [26]. However, self-control’s promotion of prosocial behavior may be moderated by friendship quality [54,55,56]. Because individuals with high friendship quality show more empathy, it causes prosocial behavior [55]. The empathy altruism model holds [54]: while other people are in trouble, onlookers will generate emotion directed at the people who need help, including compassion, compassion, empathy, etc. The greater the intensity of the feeling, the stronger the individual’s altruistic motivation to relieve others’ plights. People are more likely to engage in helping behavior. The general model of crime suggests that [55,57] individuals with low self-control have difficulty in paying attention to the needs of others and therefore, have poor friendship quality. This fragile attachment to others makes people less concerned about how others will react, which leads to crime. This suggests that friendship quality and self-control may interact to influence individual behavior. Therefore, self-control causes individuals to restrain their personal desires to help others, while individuals with high friendship quality have strong empathy and focus on the needs of others, stimulating their helping behavior. Therefore, this study hypothesized that friendship quality may play a moderating role in the relationship between self-control and prosocial behavior. Based on this, the present study proposes the following hypothesis, and the proposed theoretical model is shown in Figure 1:

Life satisfaction mediates the relationship between self-control and adolescent prosocial behavior. Friendship quality moderates the relationship between self-control and adolescent prosocial behavior.

## 2. Materials and Methods

### 2.1. Participants

The present study was approved by the Ethics Committee at Guangzhou University (No. GZHU2019012). A total of 1182 Chinese middle school students participated the survey. They were between 12 and 15 years old (average age: 14.16 years old, SD = 1.29). They were from three different middle schools (531 girls and 651 boys) in Guangzhou, Guangdong Province, China.

This study used longitudinal tracking research (T1&T2; and the interval between T1&T2 is 6 months). At T1, 1182 Chinese middle school students were asked to finish the Brief Self-Control Scale, Friendship Quality Questionnaire, Satisfaction with Life Scale, and a demographic questionnaire (including parents’ educational level, family residence, grade, age, name, gender, student number, date of birth, etc.). At T2, they were asked to finish the Prosocial Behavior Scale.

In T2, 198 participants did not complete, accounting for 10.9% of the total number of subjects. In view of the common problem of subject loss in the follow-up study, the participants who completed two tests and those who did not complete two tests were compared and analyzed (Marshall et al., 2014). The results showed that the subjects who did not complete two tests and those who completed the two tests did not differ significantly in self-control (t = 0.35, *p* = 0.954), life satisfaction (t = 0.36, *p* = 0.411), prosocial behavior (t = 0.06, *p* = 0.516), and friendship quality (t = 3.81, *p* = 0.393).

### 2.2. Procedure

The questionnaire survey adopts the method of collective test, which was conducted in the class as the unit, and is conducted by the undergraduates and postgraduates majoring in psychology. First, before the investigation, the participants gathered in a quiet environment, and the researcher in charge of the test read instructions for the survey, and the students signed their name in informed consent. After the subjects completed the questionnaire, the questionnaire was recovered on the spot, and invalid questionnaires were eliminated.

### 2.3. Brief Self-Control Questionnaire

The Brief Self-Control Questionnaire is developed by Tangney et al. [15] and was widely used to measure individual’s self-control ability. Relatively high scores indicate high levels of individual self-control. The scale showed good validity and reliability and is suitable for both Chinese and Western teenagers (middle and high schools) and adults (college students and employees). Items include “I’m good at resisting temptation” and “I’m lazy”. The higher the score, the higher the self-control tendency. All students finished the Chinese version of the Brief Self-Control Scale, which had been used in Chinese adolescent and showed good reliability and validity [26] (Dou et al., 2019). Cronbach’s α coefficient in this study was 0.86.

### 2.4. Prosocial Behavior Scale

Prosocial behavior was measured by the prosocial subscale of the Strength and Difficulties Scale. It was developed by Goodman [58] and includes five items and is a three-point scale with items such as “I often volunteer to help others (parents, teachers, classmates, etc.)” and “I often share with others (food, games, pens, etc.)”. The higher their scores, the higher the individual’s prosocial tendency; furthermore, there is no reverse score. All students finished the Chinese version of the Strength and Difficulties Scale, which had been used in Chinese adolescent and showed good reliability and validity [26]. The Cronbach’s α coefficient in this study was 0.75.

### 2.5. Friendship Quality Scale

The scale was developed by Parker and Asher [44]. It includes 18 items and is used to measure the quality of friendship quality between participants and their best friends. It is a five-point scale. The higher the overall score, the better the friendship quality. It includes items such as “We always sit together whenever we get the chance” and “We often get angry with each other”. The Cronbach’s α coefficient in this study was 0.82.

### 2.6. Life Satisfaction Scale

It was developed by Diener et al. [23] and was used to measure an individual’s life satisfaction. It is a seven-point scale. The higher the overall score, the better the level of life satisfaction. It includes items such as “My life is very full” and “I am satisfied with my life”. All students finished the Chinese version of the Satisfaction with Life Scale, which had been used in Chinese adolescent and had showed good reliability and validity [26]. Cronbach’s α coefficient was 0.85.

### 2.7. Statistical Analyses

The present study used SPSS 21.0 software for descriptive statistical analysis. In addition, mediation-moderated testing used model 5 in SPSS PROCESS V3.4 macro and was developed by Hayes [59]. Self-control, life satisfaction, friendship quality, and prosocial behavior and friendship quality were entered within the model as independent variable, mediator variable, moderator variable, and dependent variable, respectively. This study reported the effects derived from the total effect model and indirect effect.

## 3. Results

### 3.1. Common Method Deviation Test

Since the data were obtained from the same subjects, it may lead to artificial covariation between the prediction source and the criterion variable. In order to control the common method deviation, this study controls by reducing the participants’ guess of the measurement purpose, disrupting the order of the scale items, and improving the expression of the scale items. Cronbach’s α coefficient of self-control scale in this study was 0.86. The Cronbach’s α coefficient of prosocial behavior scale in this study was 0.75. The Cronbach’s α coefficient of friendship quality scale in this study was 0.82. The Cronbach’s α coefficient of life satisfaction scale in this study was 0.85. Therefore, this study has good reliability and validity. Before processing the data, this study conducted a common method deviation statistical test using Harman single-factor testing [60]. These results show the variance interpretation percentage of the first common factor is 30.76%, which is less than the critical value of 40%. It can be considered that no serious common method deviation existed.

### 3.2. Preliminary Analysis

As presented in Table 1, these results showed self-control is positively related to life satisfaction, prosocial behavior, and friendship quality. Second, life satisfaction positively correlated with prosocial behavior.

### 3.3. Mediation Testing

**Figure 2:**
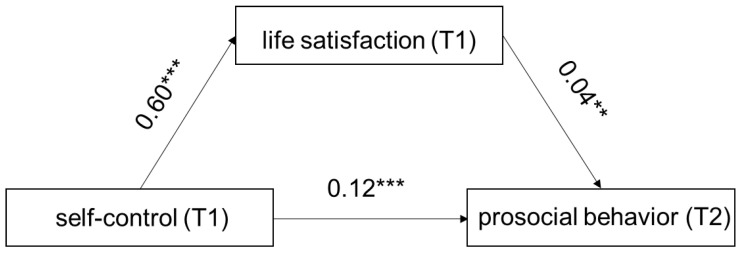
Model of the mediating role of life satisfaction between self-control and prosocial behavior. (T1) represents the score obtained at T1. (T2) represents the score obtained at T2. Note: ** *p* < 0.01, *** *p* < 0.001.

Model 4 of PROCESS Macro 3.4 (Hayes, 2013) was used to test the mediating effect. The mediation model is presented in Figure 2 After controlling for age, gender, and educational level of parents, it was determined that self-control positively predicted life satisfaction (β = 0.60, t = 9.29, *p* < 0.001, 95% confidence interval [CI] [0.47, 0.72]), and life satisfaction positively predicted the prosocial behavior (β = 0.04, t = 3.86, *p* < 0.01, 95% CI [0.02, 0.06]). Moreover, the residual effect of the self-control on the prosocial behavior was significant (β = 0.12, t = 5.35, *p* < 0.001, 95% CI [0.07, 0.16]). Bootstrapping analyses indicated that life satisfaction significantly mediated the relationship between the self-control and prosocial behavior (indirect effect = 0.02, SE = 0.01, 95% CI [0.01, 0.04]).

### 3.4. Mediation-Moderated Testing

**Figure 3:**
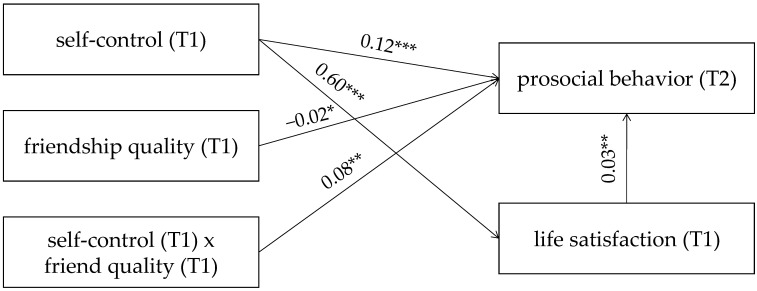
Model of the moderating role of friendship quality in the indirect relationship between self-control and prosocial behavior. (T1) represents this score obtained at T1. (T2) represents this score obtained at T2. Note: * *p* < 0.05, ** *p* < 0.01, *** *p* < 0.001.

**Figure 4:**
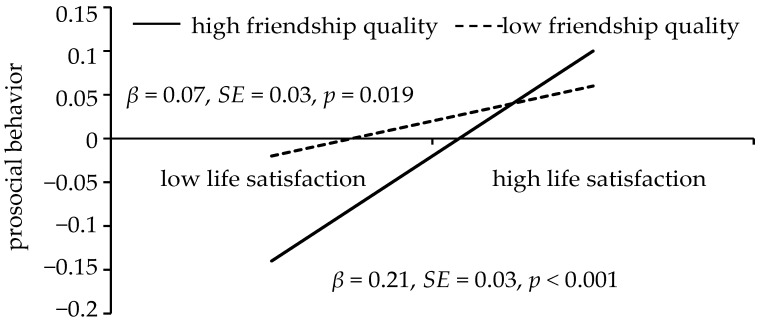
The prosocial behavior among students as a function of life satisfaction and friendship quality.

Model 5 of PROCESS Macro 3.4 (Hayes, 2013) was adopted for the mediation-moderated model testing. The mediation-moderated model was presented in Figure 3. Bias-corrected percentile bootstrap results showed that indirect effect of the self-control on the prosocial behavior through the life satisfaction is moderated by the friendship quality. Additionally, friendship quality moderated the link between self-control and prosocial behavior (β = 0.08, t = 3.20, *p* < 0.001, 95% CI [0.03, 0.13]). The present study used Model 1 of PROCESS Macro 3.4 (Hayes, 2013) to perform a simple slopes testing and determined that in Figure 4, the negative link between the self-control and prosocial behavior is significantly stronger among students with higher friendship quality (1 standard deviation above the mean; β = 0.21, t = 7.31, *p* < 0.001, 95% CI [0.16, 0.27]) than students with lower friendship quality (1 standard deviation below the mean; β = 0.07, t = 2.36, *p* = 0.019, 95% CI [0.01, 0.13]). Furthermore, the self-control is significantly positively correlated with life satisfaction (β = 0.60, t = 9.29, *p* < 0.001, 95% CI [0.47, 0.72]).

In general, bias-corrected percentile bootstrap results showed that indirect association between self-control and prosocial behavior via life satisfaction was stronger among students with high friendship quality than among those with low friendship quality. Therefore, the mediating effect of life satisfaction between self-control and prosocial behavior is moderated by friendship quality.

## 4. Discussion

The present results enhanced our understanding of how self-control influences adolescent prosocial behavior and also contribute to crucial targets for improving adolescents prosocial behavior.

This result indicated that life satisfaction mediated the link between self-control and adolescent prosocial behavior. This may be because self-control strategies adopted by highly self-controlled individuals may be more effective, which are conducive to the advancement and realization of individual goals. In this process, students with high self-control ability experience more life satisfaction and happiness [61]. Because individuals who use more self-control strategies can actively seek available resources to obtain desired goals, and they were more likely to solve conflict dilemmas, they therefore experience more life satisfaction. Meanwhile, life satisfaction causes individuals to produce positive emotions [62]. In the process of intercommunication, individuals regard positive emotions as information promoting interpersonal communication [63] In terms of the nature of positive emotions, positive emotions are also related to prosocial behavior. Therefore, individuals with high life satisfaction have more interpersonal communication, which helps in strengthening individual social bonds and producing more social behavior [64].

Our results were consistent with the hypothesis that friendship quality moderates the relationship between self-control and adolescent prosocial behavior. First, for adolescents with high friendship quality, prosocial tendency was higher than that of those students with low level friendship quality. It is easy to understand because students with high-level friendship quality tend to show more empathy and therefore were more likely to understand the needs of other people and showed more prosocial behavior [52,53]. Second, friendship quality could significantly enhance the effect of self-control on adolescent prosocial behavior. This is mainly because individuals with high friendship quality have stronger empathy and positive emotion, which can promote prosocial behavior, while prosocial behavior requires self-control to suppress impulsive responses to short-term benefits. Previous research has found that positive emotions help enhance prosocial behavior [65,66,67]. McCullough et al. [68] believed that the function of positive emotions promotes reciprocal altruistic behaviors. Although prosocial behavior may damage individual interests in the short term, the establishment of reciprocal relationships can enhance individual long-term interests; thus, it has evolutionary advantages. Previous studies also showed that positive emotions can improve self-control [69,70]. Our results showed that friendship quality plays an important role in link between self-control influencing prosocial behavior.

This study has several educational implications. First, for individuals, the quality of peer affiliation significantly influences the shaping of individuals’ thoughts and behaviors. Affiliation with high-quality peers is beneficial to individuals’ social adaptation and physical and mental health development. Conversely, deviant peer affiliations make adolescents more likely to learn problematic behavior from their peers (such as smoking, drinking, and fighting), which reduces their self-control and is not beneficial for future social adaptation. Therefore, it is important for adolescents to consciously choose their peers and integrate them into a positive and harmonious group of peers. Second, the contact between adolescents and their peers becomes increasingly close during adolescence, and the influence of peers cannot be ignored. While giving children freedom and space to make friends, parents should also pay attention to the quality of interpersonal interactions. Through encouragement and supportive suggestions, parents should encourage teenagers to choose high-quality friends and participants in positive and beneficial social activities.

There are some limitations to our study. Firstly, the present study used a longitudinal tracking survey and avoided the weakness of a cross-sectional study. The present study revealed that self-control affects satisfaction, and satisfaction affects prosocial behavior. Only two time points were explored in this study. Future studies may adopt more time points to further explore the link between self-control and prosocial behavior. Second, the data of this study came from the self-reports of middle-school students. Future research should collect data with relatively more comprehensive methods (observation, questionnaire, and interview) and multiple channels (self-report, teacher reports, parent reports, and peer reports) to conduct a more comprehensive study. Current research focuses on the influence of self-control, life satisfaction, and friendship quality on prosocial behavior. The PROCESS macro in SPSS provided a good answer to this question. Future research can use a variety of models to explore this problem. Finally, the finding of this survey should be extended to other samples (college students) for extensive testing.

## 5. Conclusions

By exploring a mediation moderated model, the present research revealed how high self-control ability will increase prosocial behavior in adolescents. Overall, high self-control ability can also promote adolescents’ life satisfaction, which in turn can increase prosocial behavior. Moreover, the conducive effect of self-control may be strengthened by a high-level friendship quality.

## Figures and Tables

**Table 1:** Correlations and descriptive statistics for all of variables.

Variables	1	2	3	4
1. Self-control				
2. Life Satisfaction	0.31 ***			
3. Prosocial Behavior	0.21 ***	0.16 ***		
4. Friendship Quality	0.18 ***	−0.05	0.01	
*Mean*	3.13	4.26	2.44	2.87
*SD*	0.56	1.28	0.41	0.84

Note: *** *p* < 0.001.

## Data Availability

The data presented in this research can be made available from the correspondence author.
